# Impact of elotuzumab treatment on pain and health-related quality of life in patients with relapsed or refractory multiple myeloma: results from the ELOQUENT-2 study

**DOI:** 10.1007/s00277-018-3469-4

**Published:** 2018-09-04

**Authors:** David Cella, Jan McKendrick, Amber Kudlac, Antonio Palumbo, Abderrahim Oukessou, Ravi Vij, Teresa Zyczynski, Catherine Davis

**Affiliations:** 1 Northwestern University, Chicago, IL USA; 2 PRMA Consulting Ltd, Fleet, Hampshire, UK; 3 University of Technology Sydney, Ultimo, NSW Australia; 4 University of Turin, Turin, Italy; 5 Bristol-Myers Squibb, Princeton, NJ USA; 6 Washington University School of Medicine, St. Louis, MO USA

**Keywords:** Pain, Health-related quality of life, Patient-reported outcomes, Multiple myeloma

## Abstract

Treatment of relapsed/refractory multiple myeloma (RRMM) aims to prolong survival while maintaining health-related quality of life (HRQoL) by managing disease-related symptoms and complications—one of the most frequent and debilitating being bone pain. In the ELOQUENT-2 study (NCT01239797), which evaluated the addition of elotuzumab to lenalidomide plus dexamethasone versus lenalidomide plus dexamethasone, pain and HRQoL were assessed in patients with relapsed/refractory disease using the Brief Pain Inventory–Short Form (BPI-SF) and the European Organisation for Research and Treatment of Cancer (EORTC) Quality of Life Questionnaire–Core 30 module (QLQ-C30) and myeloma-specific module (QLQ-MY20). Mean baseline pain scores were low and remained so throughout treatment with both regimens; mean HRQoL scores did not change substantially from baseline. A significantly higher proportion of patients with objective response than without had clinically meaningful improvements in worst pain over two consecutive treatment cycles (29 versus 12%; *p* < 0.001). Patients with very good partial response (VGPR) or better reported reduced scores for pain severity and worst pain; those with progressive disease reported increased scores for these domains and pain interference. These findings show that previously reported improvements in progression-free survival and response rate with elotuzumab are achieved without detriment to HRQoL, which is maintained over time.

**Electronic supplementary material:**

The online version of this article (10.1007/s00277-018-3469-4) contains supplementary material, which is available to authorized users.

## Introduction

Multiple myeloma (MM), considered incurable, accounts for 10% of hematologic malignancies [1]. The median age at diagnosis is 69 and 72 years in the USA and Europe, respectively [2, 3], and a significantly shorter median survival is observed in patients ≥ 50 years of age [4]. Immunomodulatory agents and proteasome inhibitors have increased response rates and progression-free survival (PFS). However, most patients eventually experience relapse or develop refractory disease [5–8]. Current therapies aim to maintain health-related quality of life (HRQoL) while prolonging survival. Improved survival and consequent lengthening of the disease course is associated with an increased burden of disease- and treatment-related symptoms. One of the most frequent symptoms of MM impairing QoL is bone pain [9–14]. Furthermore, increasingly complex/aggressive therapies are associated with an increased risk of treatment-related toxicities.

Demonstrating improvements in HRQoL is challenging in patients with advanced or chronic diseases as patients’ perceptions of their health status and responses to HRQoL-related questions can be affected by adaptations to the disease [15, 16]. In the absence of actual HRQoL improvements, HRQoL preservation during treatment may be seen as a benefit.

An informed treatment decision requires an adequate understanding of the potential benefits and risks by both physician and patient. When treatments offer a clear survival benefit but have increased toxicity, it is important to consider the patient’s experience of treatment and HRQoL. In order to inform benefit-risk assessments, it is essential to use validated, disease-relevant, patient-reported outcome (PRO) measures in clinical studies to assess symptoms, functioning (activity limitations), health status/HRQoL, patient satisfaction, treatment preferences, and adherence.

Elotuzumab, an immunostimulatory antibody against signaling lymphocytic activation molecule F7 (SLAMF7), is indicated in combination with lenalidomide and dexamethasone (Ld) for the treatment of MM in patients who have received one to three prior therapies in the US [17] or at least one prior therapy in Europe [18]. In the phase 3 ELOQUENT-2 study (NCT01239797), elotuzumab in combination with Ld (ELd) was compared with Ld in patients with relapsed and/or refractory MM (RRMM) [19]. ELd improved PFS, reducing the risk of disease progression or death by 30% versus Ld (hazard ratio = 0.70; 95% confidence interval 0.57, 0.85; *p* < 0.001) [19]. PFS was sustained over time, with a relative improvement of 44% for ELd versus Ld at 3 years [20] and 50% at 4 years [21]. Overall response rate (ORR) was also improved at 3- and 4-year follow-up (79% with ELd versus 66% with Ld at both analyses) [20, 21]. Patients with a very good partial response (VGPR) or better (International Myeloma Working Group [IMWG] Uniform Response Criteria for Multiple Myeloma definition) [22, 23] achieved longer PFS than those with minimal response or stable disease following treatment with ELd [19], consistent with prior reports regarding depth of response and survival [22–24]. ELd had a similar safety profile and discontinuation rate to Ld.

In ELOQUENT-2, PRO measures (Brief Pain Inventory–Short Form [BPI-SF], European Organisation for Research and Treatment of Cancer [EORTC] Quality of Life Questionnaire–Core 30 module [QLQ-C30] and myeloma-specific module [QLQ-MY20]) were included as pre-specified secondary or exploratory endpoints. In this paper, we investigated HRQoL measures and whether there is a relationship between treatment response and patient-reported pain. These analyses, performed using data from the extended 3-year follow-up, aim to provide patients and physicians with a more comprehensive benefit-risk assessment of elotuzumab in RRMM.

## Methods

### Study design and patients

The ELOQUENT-2 study design has been previously described (see Online Resource: Methods). Institutional review board or independent ethics committee approval and written informed consent were obtained. All patients had received one to three prior therapies and had disease progression after their most recent therapy [19].

The PRO population comprised all randomized patients with a baseline assessment and at least one follow-up PRO assessment. Pain and HRQoL were assessed using the BPI-SF, EORTC QLQ-C30, and QLQ-MY20 at screening, on day 1 of each 28-day cycle, and at the end of treatment. Change from baseline in mean BPI-SF scores for pain severity and pain interference was pre-specified secondary endpoints of ELOQUENT-2 (Online Resource: Methods, Table 1).

### Definition of meaningful and clinically relevant differences

In the current study, a mean change from baseline of ≥ 10 points in the EORTC QLQ-C30 and QLQ-MY20 domain scores was considered meaningful [25–27].

Although a minimally important difference of 2 points has been established for the BPI-SF worst pain for breast cancer [28], it has not been reported in MM, and therefore, we estimated MM-specific meaningful differences for worst pain, pain severity, and pain interference using distribution-based methods (Online Resource: Methods, Table 2) [29].

Threshold levels were used to interpret the clinical relevance of treatment differences in mean EORTC QLQ-C30 scores (previously published threshold values for trivial, small, medium, and large clinically relevant differences for each domain [Online Resource: Methods, Table 3]) [30]. Although not specific to an MM population, they were used to indicate potential clinically relevant differences in this study.

### Pain response

Group- and patient-level pain data were assessed. A meaningful pain response for worst pain was defined as a 30% reduction from baseline score (equivalent to a change of ≥ 2–3 points on the 11-point BPI-SF scale, depending on the starting value) [31, 32]. In rate-of-response analyses, this level of response sustained for two consecutive cycles while on treatment was considered a clinically meaningful pain response. These response criteria were applied to pain severity and pain interference.

### Statistical analyses

Statistical analyses were pre-specified. Descriptive statistics were generated to describe outcomes at each time point for the absolute value and calculated change from baseline. Absolute values were compared with baseline within each treatment group (paired *t* test) and between groups (unpaired *t* test). Hypothesis testing was at the 5% (two-sided) significance level.

The overall pain response rate (chi-squared test) and time to sustained response (log-rank test) were compared between treatments (see Online Resource: Methods for further information).

## Results

### Patient population

The study population baseline characteristics have been described previously [19]. In total, 646 patients were randomized: 321 received ELd and 325 Ld (Online Resource: Fig. 1). Of these, 319 ELd-treated and 311 Ld-treated patients had at least one post-baseline PRO assessment and were included in PRO analyses. Baseline scores for the BPI-SF, EORTC QLQ-C30, and QLQ-MY20 domains were comparable between treatments. Questionnaire completion rates at baseline and end of treatment were similar between the ELd and Ld groups (90 and 92% at baseline; 61 and 62% at end of treatment, respectively), remained greater than 65% until cycle 40, and then decreased (due to limited numbers of eligible patients). A lower proportion of ELd- versus Ld-treated patients discontinued treatment (64.2% [206/321] versus 76.9% [250/325]). The maximum number of ELd treatment cycles was 42 and 40 for Ld. As small sample sizes in the later cycles may have reduced robustness, results are reported for cycles with ≥ 30 patients.

**Fig. 1:**
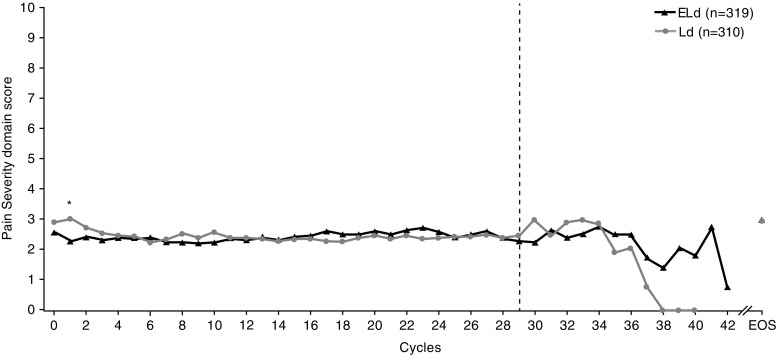
Patient-reported pain severity during treatment: mean absolute values by treatment. The dashed line indicates < 30 patients per treatment group. *ELd* elotuzumab, lenalidomide, and dexamethasone, *EOS* end of study visit, *Ld* lenalidomide and dexamethasone

### Patient-reported pain during treatment

Mean baseline scores for the pain severity domain of the BPI-SF were low for both ELd and Ld arms (2.6 versus 2.9) and were equivalent to a “ mild pain” rating [33]. Baseline values were also low for the pain interference and worst pain domains. Mean scores for all domains did not change to any great extent and remained similar between treatments over the course of the study (Fig. 1; Online Resource: Fig. 2). The change from baseline in pain severity, pain interference, and worst pain scores was minimal in both groups up to cycle 30, and was below preset estimates of minimally important differences. Beyond cycle 30, population size was too small for robust analysis.

These observations were supported by the pre-specified mixed-model repeated-measures (MMRM) analyses, which showed very small average increases in pain scores (mean increases of 0.717–1.121 for ELd and 0.673–0.998 for Ld) across the three domains; the difference between treatments was not statistically significant for any domain (Online Resource: Table 4).

For all three BPI-SF domains, the MMRM models with covariates showed that baseline scores had a significant relationship with pain outcomes: higher baseline scores were associated with greater change from baseline. The models also predicted that several patient and clinical characteristics could influence patients’ experience of pain and result in a greater mean change from baseline. These factors were Eastern Cooperative Oncology Group (ECOG) performance status (for a performance status of 0 or 1 versus 2), and age (for age ≥ 65 versus < 65 years) and prior stem cell transplantation (with versus without prior transplantation) in the pain severity and worst pain models.

**Fig. 2:**
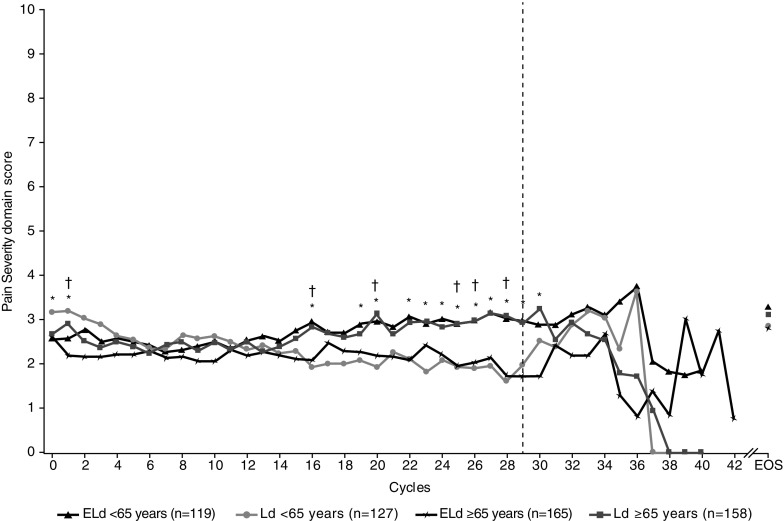
Patient-reported pain severity during treatment, by age group: mean absolute values in patients < 65 and ≥ 65 years of age. The dashed line indicates < 30 patients per treatment group. Asterisks (*) denote statistical significance (*p* < 0.05) for the difference between treatments in the <65 years age group; daggers (†) denote statistical significance (*p* < 0.05) for the difference between treatments in the ≥65 years age group. *ELd* elotuzumab, lenalidomide, and dexamethasone, *EOS* end of study visit, *Ld* lenalidomide and dexamethasone

**Fig. 3:**
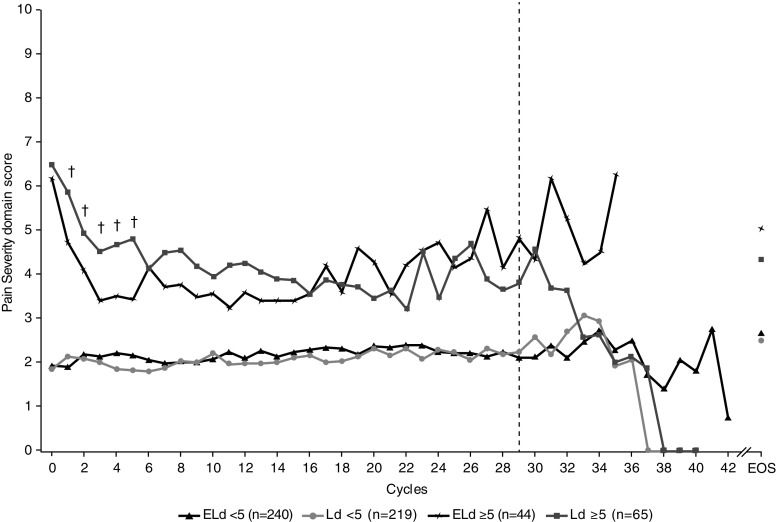
Patient-reported pain severity during treatment, by baseline score: mean absolute values in patient subgroups with (score ≥ 5) or without (score < 5) moderate/high pain at baseline. The dashed line indicates < 30 patients per treatment group. Daggers (†) denote statistical significance (*p* < 0.05) for the difference between treatments in the subgroup with a pain severity score ≥ 5. *ELd* elotuzumab, lenalidomide, and dexamethasone, *EOS* end of study visit, *Ld* lenalidomide and dexamethasone

Subgroup analyses showed that age (≥ 65 versus <65 years) and severity of pain at baseline (pain severity score of ≥ 5 versus < 5) influenced patient-reported pain. Mean pain severity scores in the older age group were lower with ELd versus Ld; the differences between treatments were statistically significant at cycles 1, 16, 20, 25, 26, and 28. In the younger age group, mean scores were also slightly lower with ELd than with Ld; differences between treatments were statistically significant at cycles 1, 16, 19, 20, and 22–30 (Fig. 2). Despite small numbers of patients with moderate-to-severe pain (pain severity score ≥ 5) at baseline, there were statistically significant differences between treatments in mean scores at cycles 1–5 (Fig. 3). Similar patterns were seen for pain interference and worst pain domains (data not shown).

### Rate and time to sustained improvement in worst pain response

**Fig. 4:**
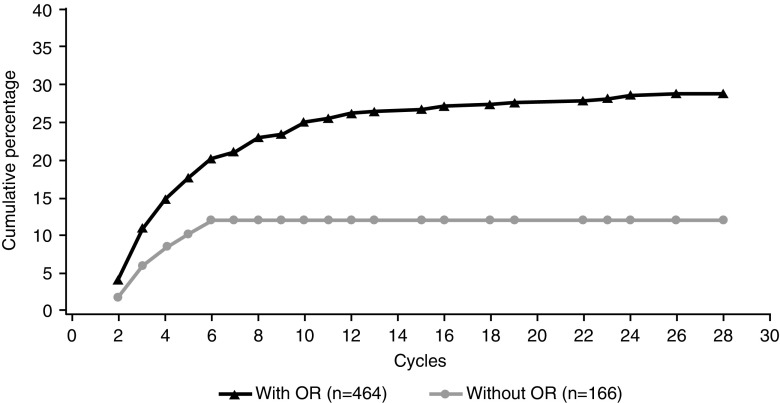
Pain response during treatment, by clinical response: cumulative percentage of patients with a sustained improvement in worst pain among those who achieved, or did not achieve, an objective response to treatment in the overall population. *OR* objective response

A similar proportion of ELd- and Ld-treated patients showed sustained improvement in worst pain during treatment (26 versus 24%; Online Resource: Table 5); there were no statistically significant differences in time to achieving a pain response. Pain responses were generally achieved early in treatment; few occurred after cycle 10 (Fig. 4).

The cumulative percentage of patients across both groups who had a pain response for worst pain was significantly higher in those with versus those without an objective response (OR) to treatment (29 versus 12%; *p* < 0.001; Fig. 4; Online Resource: Table 5). There were no significant differences in time to achieving pain response between patients with and without an OR (Fig. 4). There were no significant differences between treatments in the cumulative proportion of patients with a sustained improvement in worst pain, or in time to sustained improvement in the subgroups based on OR and treatment received (Online Resource: Fig. 3).

### Pain response by best response to treatment

**Fig. 5:**
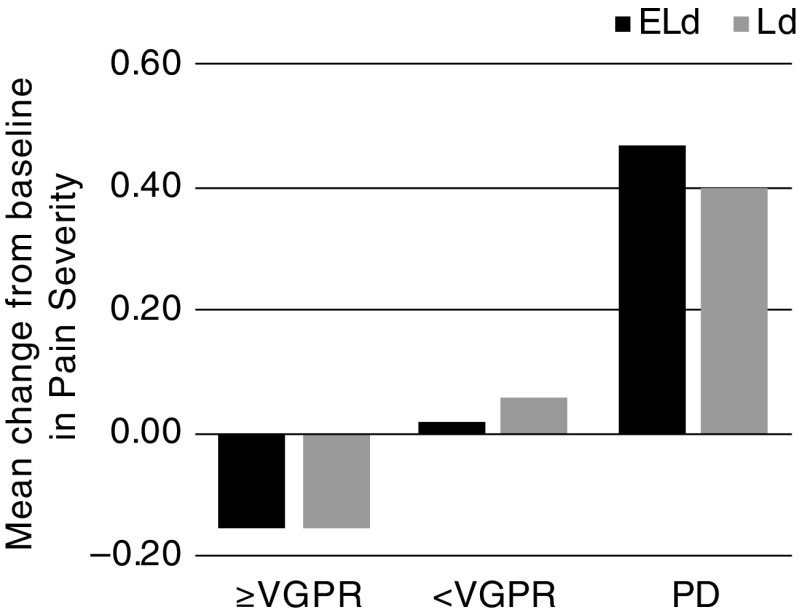
Patient-reported pain during treatment, by best response to treatment: mean change from baseline in pain severity in patients with a best response to treatment of at least a VGPR, less than a VGPR, or progressive disease. *ELd* elotuzumab, lenalidomide, and dexamethasone, *Ld* lenalidomide and dexamethasone, *PD* progressive disease, *VGPR* very good partial response

Mean change from baseline in worst pain, pain severity, and pain interference scores was analyzed for subgroups based on best response to treatment according to the IMWG Uniform Response Criteria for Multiple Myeloma (Online Resource: Methods) [22, 23]. Changes for all three domains were similar for ELd and Ld for each level of best response to treatment: patients with progressive disease reported an increase from baseline; those with a VGPR or better reported a reduction from baseline in mean pain severity and worst pain, but not in pain interference (**Fig.** 5; Online Resource: Fig. 4); patients with less than a VGPR but no disease progression showed an overall mean change from baseline intermediate between that of patients with a VGPR or better, and those with progressive disease.

### HRQoL assessment

#### EORTC QLQ-C30

There was no decrement in HRQoL—mean values for the key domains of fatigue, physical functioning, global health status/QoL, and pain remained relatively unchanged over time in either treatment arm. There were trends towards improvement in HRQoL for ELd versus Ld, with mean values generally lower for fatigue and pain and higher for physical functioning and global health status/QoL with ELd (Online Resource: Fig. 5). Clinically relevant between-treatment group differences for these domains were observed at a few time points (Online Resource: Fig. 5). No changes > 10 points occurred with either treatment before cycle 30.

The MMRM analyses showed no statistically significant difference between ELd and Ld for any of these domains (Table 1).

**Table 1:** MMRM model with covariate estimate of overall treatment effect for key EORTC QLQ-C30 domains

Domain	Treatment	Estimate of effectMean (SE)	Estimated differenceMean (95% CI)	*p* value^a^
Fatigue^b^	ELd	12.228 (4.7816)	0.674 (− 3.854, 2.506)	0.6772
	Ld	12.902 (4.6763)		
Physical functioning^c^	ELd	8.096 (4.0206)	1.308 (− 4.053, 1.437)	0.3497
	Ld	6.788 (3.9393)		
Global health status/QoL^c^	ELd	3.547 (3.8702)	0.460 (− 3.020, 2.100)	0.7241
	Ld	3.087 (3.7918)		
Pain^b^	ELd	3.308 (5.3249)	1.651 (− 5.198, 1.895)	0.3608
	Ld	4.689 (5.2041)		

^a^Difference between treatment groups in overall change from baseline; ^b^Decrease reflects reduction in symptom burden; ^c^Increase reflects improved functioning. *CI* confidence interval, *ELd* elotuzumab, lenalidomide, and dexamethasone, *EORTC QLQ-C30* European Organisation for Research and Treatment of Cancer Quality of Life Questionnaire–Core 30 module, *Ld* lenalidomide and dexamethasone, *MMRM* mixed-model repeated-measures, *QoL* quality of life, *SE* standard error

#### EORTC QLQ-MY20

Mean values for the domains of disease symptoms and side effects of treatment were similar for treatment groups and remained relatively stable over time (Online Resource: Fig. 6). MMRM analyses showed no statistically significant differences between treatments for the change from baseline in these key EORTC QLQ-MY20 domains (Table 2). Meaningful changes from baseline (≥ 10 points) were observed only at cycles where analyses were considered unreliable (< 30 patients per treatment group).

**Table 2:** MMRM estimate of overall treatment effect for key EORTC QLQ-MY20 domains (covariate model)

Domain	Treatment	Estimate of effectMean (SE)	Estimated differenceMean (95% CI)	*p* value^a^
Disease symptoms^b^	ELd	7.274 (3.8658)	− 0.817 (− 3.377, 1.742)	0.5307
	Ld	6.457 (3.7718)		
Side effects of treatment^b^	ELd	3.441 (2.8190)	− 0.324 (− 2.201, 1.552)	0.7345
	Ld	3.765 (2.7524)		

^a^Difference between treatment groups in overall change from baseline; ^b^Decrease reflects reduction in symptom burden. *CI* confidence interval, *ELd* elotuzumab, lenalidomide, and dexamethasone, *EORTC QLQ-MY20* European Organisation for Research and Treatment of Cancer Quality of Life Questionnaire–myeloma-specific module, *Ld* lenalidomide and dexamethasone, *MMRM* mixed-model repeated-measures, *SE* standard error

## Discussion

ELOQUENT-2 pre-specified PRO and HRQoL endpoints allowed us to assess the effect of elotuzumab in combination with Ld on HRQoL and patient-reported pain, identify predictors of improvements in pain and HRQoL during treatment, and examine the relationship between best response to treatment and patient-reported pain.

Adding elotuzumab to Ld did not cause a decrement to key HRQoL domains or a clinically relevant increase in patient-reported pain. Pain levels were low at baseline, consistent with a “mild pain” rating [33], and only 9% of patients had a poor ECOG performance status of 2 [19]. These low levels of pain were maintained during treatment with both ELd and Ld and may have limited further reductions in pain severity. This is supported by our observation that changes in mean pain scores were greater in patients with moderate-to-severe pain at baseline. The combination of low pain levels at baseline and the high thresholds for improvement used in this study (in comparison with other MM studies [34]) meant that demonstrating clinically meaningful improvement in pain was challenging. It was therefore not surprising that differences in other domains did not emerge.

Data presented here demonstrate that differences in the pain experience occurred early in treatment (cycles 1 to 2); however, differences between treatments also emerged in later cycles, in subgroups based on age. In several patients with vertebral compression fractures, it took several months for the pain to improve. Although these results are surprising and possibly due to the heterogeneity in the data, the pattern was consistent across several pain endpoints (BPI-SF domains and pain domain of the EORTC QLQ-C30).

Addition of a new therapeutic agent to an existing single or double regimen may improve clinical outcomes without compromising HRQoL, as observed with the ELd combination in RRMM, and with other double and triple combinations in metastatic melanoma and metastatic pancreatic ductal adenocarcinoma [35, 36]. Treatment may therefore be maintained over a longer period, with potential survival benefits.

The quality of PRO data collected during clinical studies is improving, as are the analyses and reporting of these data; however, there are still elements, such as dosing convenience, that may affect HRQoL but are not adequately measured by current PRO instruments [37].

PRO data are important to demonstrate the effects of cancer and its treatment on patients’ lives; identify patients who may benefit the most from a specific therapy; and inform clinicians about the relative benefits and risks of its efficacy, toxicity, and value from the patient perspective [38]. Several regulatory and clinical research organizations have ongoing initiatives to develop frameworks for improving benefit-risk assessment and PRO measures, as there is no widely accepted method of benefit-risk quantification [37, 39–41]. High-quality PRO data will feed into future benefit-risk analyses.

Our robust, scientifically valid methodology addressed many issues common to analyses of PRO data, particularly regarding missing data. Following a pre-defined statistical analysis plan, data were analyzed from all PRO and HRQoL domains included in the study. Validated and disease-specific PRO instruments were used to measure outcomes important to patients with RRMM. We focused on the experience of pain during treatment, as patients with MM have reported that this significantly impairs HRQoL [25], and conducted secondary analyses to assess the influence of demographic/clinical factors on patients’ perceptions of the effect of MM or its treatment on HRQoL.

Data from the ASPIRE study [34] showed comparable levels of baseline HRQoL, as measured by the global health status/QoL domain of the EORTC QLC-C30, to those reported in ELOQUENT-2. Mean values for the ELd group (ELOQUENT-2) and carfilzomib group in ASPIRE were comparable at equivalent time points (cycles 1, 3, 6, 12, and 18), as was the magnitude of benefit seen with each treatment, although applying a lower threshold for meaningful improvement in ASPIRE (5 points) than in ELOQUENT-2 (10 points) may have implied some impact on HRQoL. Results from ELOQUENT-2 demonstrated that HRQoL is sustained in a number of domains important to patients; this was seen over an extended period of follow-up, with greater differences in certain subgroups.

This study has some limitations. The open-label trial design may have influenced investigators’ and patients’ treatment expectations during self-reported assessments. However, it is worth noting that, according to the study protocol, response was evaluated every 4 weeks from date of first dose of study drug until disease progression, death, or withdrawal of consent. Therefore, the differences seen between treatment groups in terms of patient-reported pain response at cycle 2 of treatment (Online Resource: Fig. 3) between patients with versus those without an objective response occurred at the time that the first evaluation scans were performed. As such, these PRO data seem to support a reduction in pain owing to treatment response that at this time point would not have been influenced by either patient or investigator knowledge of first evaluation scan results. Study discontinuations leading to missing data can complicate the interpretation of PRO and HRQoL results, and this study had lower completion rates at the end of treatment compared with baseline. However, additional statistical analyses were performed to compensate for this.

## Conclusions

This study shows that the previously reported improvements in PFS and ORR with elotuzumab in patients with RRMM [19] were achieved without meaningful detriment of pain or to HRQoL, and that pain scores and HRQoL were maintained over time. Treatment responders showed more HRQoL and pain benefit than non-responders, supporting the clinical relevance of PROs in MM care.

## Electronic supplementary material

ESM 1 (PDF 1030 kb)
