# Supplementary material for: Impact of elotuzumab treatment on pain and health-related quality of life in patients with relapsed or refractory multiple myeloma: results from the ELOQUENT-2 study
Source: Ann Hematol. 2018 Sep 4;97(12):2455–63. doi: 10.1007/s00277-018-3469-4 (PMC6208683; doi:10.1007/s00277-018-3469-4)
Supplement: Supplementary file 1 — (PDF 1030 kb) [file 277_2018_3469_MOESM1_ESM.pdf]

Online Resource

Journal: *Annals of Hematology*

Impact of elotuzumab treatment on pain and health-related quality of life in patients with relapsed or refractory multiple myeloma: results from the ELOQUENT-2 study

David Cella • Jan McKendrick • Amber Kudlac • Antonio Palumbo • Abderrahim Oukessou • Ravi Vij • Teresa Zyczynski • Catherine Davis

Corresponding author:

Jan McKendrick

PRMA Consulting Ltd, Linea House, Harvest Crescent, Ancells Business Park, Fleet GU51 2UZ, Hampshire, UK

Telephone: +44 1252 786284

Email: jmckendrick@prmaconsulting.com

Methods

ELOQUENT-2 study design

ELOQUENT-2 was conducted in accordance with the Helsinki Declaration of 1975 and was approved by the institutional review board or independent ethics committee at each study site before initiation. Written informed consent was obtained from all patients. ELOQUENT-2 was a phase 3 open-label trial involving patients ≥18 years of age with relapsed and/or refractory multiple myeloma (RRMM) and measurable disease. All patients had received one to three prior therapies and had disease progression after their most recent therapy. No more than 10% of patients had received lenalidomide therapy.

Patients received either elotuzumab in combination with lenalidomide and dexamethasone (ELd) or lenalidomide and dexamethasone (Ld) alone in 28-day cycles until disease progression or unacceptable toxicity.

Patient-reported outcome (PRO) measures for pain and health-related quality of life (HRQoL)

The Brief Pain Inventory–Short Form (BPI-SF) is an 11-item measure from which summary measures are reported: Pain Severity (based on four items: worst, least, average, and current pain), Pain Interference (based on seven items: general activity, mood, walking, normal work, relations with others, sleep, and enjoyment of life), and Worst Pain (single item). For each item, patients report their pain and its impact during the preceding 24-hour period using an 11-point numeric scale (0–10); in all cases, lower scores represent a better health state (Online Resource: Table 1) [1, 2]. In the current study, Worst Pain, Pain Severity, and Pain Interference were scored in accordance with the scoring manual [3].

The European Organisation for Research and Treatment of Cancer Quality of Life Questionnaire–Core 30 module (EORTC QLQ-C30) has 30 items grouped into 15 domains (five functional domains, nine symptom domains, and one Global Health Status/quality of life [QoL] domain). All items have four response options (except for Global Health Status/QoL, which has seven options) for patients to report their state of health at a given point [3]. The EORTC–Myeloma-specific module (EORTC QLQ-MY20), a supplement to the EORTC QLQ-C30 for patients with multiple myeloma (MM), has 20 items grouped into four domains: Disease Symptoms, Side Effects of Treatment, Future Perspective, and Body Image (Online Resource: Table 1). All items have four response options for the patient to report myeloma-specific symptoms or

problems during the previous week [4]. In the current study, HRQoL measures were scored in accordance with the manual [3].

Table 1
Summary of the key domains of the BPI-SF, EORTC QLQ-C30, and EORTC QLQ-MY20

Instrument	Domains of interest	Range	Interpretation
BPI-SF	Pain Severity	0–10	Higher score indicates a worse outcome
	Pain Interference		
	Worst Pain		
EORTC QLQ-C30	Physical Functioning	0–100	Higher score indicates better functioning
	Fatigue		Higher score indicates worse fatigue
	Global Health Status/QoL		Higher score indicates better health status/QoL
	Pain		Higher score indicate worse pain
EORTC QLQ-MY20	Side Effects of Treatment		Higher score indicates worse disease symptoms and side effects of treatment
	Disease Symptoms		

BPI-SF Brief Pain Inventory–Short Form, *EORTC QLQ-C30* European Organisation for Research and Treatment of Cancer Quality of Life Questionnaire–Core 30 module, *EORTC*

QLQ-MY20 European Organisation for Research and Treatment of Cancer Quality of Life Questionnaire–Myeloma-specific module, *QoL* quality of life

Handling of missing and incomplete data for the PRO instruments

Mean Pain Interference scores and mean HRQoL domain scores were calculated if at least 50% of the items that construct the domain had been answered. If more than 50% of the items were missing, the domain score was considered missing. The mean Pain Severity score was calculated based on all four severity items; missing items were not imputed at an individual patient level [2]. Thus, the score was not calculated if any items were missing.

Missing domain-level data or data for the entire instrument were assumed to be missing at random; no adjustments were made to account for any missing domains or assessments.

Meaningful change from baseline

The minimal important difference (MID) is the smallest difference in the domain of interest that patients consider beneficial and that would mandate a change in the patient's management, in the absence of troublesome side effects and excessive cost [5]. The MID needs to be met or exceeded at the group level in order for reported changes to be considered meaningful. The MID defines meaningful change for patients and should not be interpreted as clinically relevant. The method of calculation of MID thresholds for the BPI-SF, EORTC QLQ-C30, and QLQ-MY20 are described below.

BPI-SF

An MID has not been established for the BPI-SF in MM. Distribution-based methods were used to calculate MM-specific MIDs for the Worst Pain item and the Pain Severity

and Pain Interference baseline scores using two methods: one based on standard deviation (SD), and the other based on standard error of the mean (SEM) (Online Resource: Table 2) [6, 7]. The SEM method was not applied to Worst Pain as this is a single item and internal consistency cannot be tested. For Pain Severity and Pain Interference, the mean change from baseline for each treatment group at each cycle was compared with these two calculated MID thresholds; for Worst Pain, the comparison was with the SD-based method only.

Table 2
MID thresholds for key domains of the BPI-SF

BPI-SF domain	Method 1: 0.5 SD	Method 2: –1 SEM
Pain Severity	1.148<br>(n=568)	–0.564<br>(n=568)
Pain Interference	1.278<br>(n=565)	–0.586<br>(n=559)
Worst Pain	1.451<br>(n=574)	–[a]

[a]Method 2 could not be used for Worst Pain as it is a single-item score. *BPI-SF* Brief Pain Inventory–Short Form, *MID* minimal important difference, *SD* standard deviation, *SEM* standard error of the mean

EORTC QLQ-C30 and QLQ-MY20

For the purpose of interpreting changes in the EORTC QLQ-C30 scores from baseline, a mean change of at least 10 points on the standardized domain scores was required for

the change to be considered meaningful (i.e., the MID), reflecting the lower benchmark for a moderate change [8]. Similarly, for the purpose of interpreting changes in EORTC QLQ-MY20 scores from baseline, a mean change from baseline of at least 10 points on the standardized domain scores was required for the change to be considered meaningful, reflecting the highest published MID for a QLQ-MY20 domain (Disease Symptoms), and to facilitate comparison with the QLQ-C30 [6].

Clinically relevant between-group differences

Threshold levels can be used to interpret the clinical relevance of treatment group differences in mean EORTC QLQ-C30 scores. Threshold values for trivial, small, medium, and large mean differences between treatment groups have been published for each domain (Online Resource: Table 3) [9]. These values are not specific to the MM population, but were used in the current study to indicate potential treatment group differences that may be clinically relevant. As there is no clear guidance on use of the actual thresholds, any value greater than the maximum rounded value of the trivial range was considered to indicate a clinically relevant difference between treatments. The clinically relevant thresholds were not applied to subgroup analyses or to cycles in which there were fewer than 30 patients per treatment group.

Table 3
Published threshold values for mean between-group differences for key domains of the EORTC QLQ-C30

Domain	Trivial	Small	Medium	Large	Clinically relevant
Fatigue	0–5	5–13	13–19	>19	>5.0

Physical Function	0–5	5–14	14–22	>22	>5.0
Global Health Status/QoL	0–4	4–10	10–15	>15	>4.0
Pain	0–6	6–13	13–19	>19	>6.0

EORTC QLQ-C30 European Organisation for Research and Treatment of Cancer Quality of Life Questionnaire–Core 30 module, *QoL* quality of life

Mixed-model repeated-measures (MMRM) analyses of the BPI-SF domains

Pre-specified MMRM analyses were used to estimate the extent of the difference between treatments in terms of change from baseline in Pain Severity and Pain Interference scores. Each model included all cycles for which data were available for at least 30 patients in both treatment groups. An expanded MMRM model was also developed to include a number of covariates: age, sex, Eastern Cooperative Oncology Group performance status, prior stem cell transplantation, high-risk genetics, time from diagnosis, extramedullary plasmacytoma, baseline creatinine clearance, number of prior lines of treatment, and depth of response. This expanded MMRM model was used to analyze the Pain Severity, Pain Interference, and Worst Pain data.

Additional post-hoc analyses with these models compared the treatment in terms of the change from baseline in the EORTC QLQ-C30 and QLQ-MY20 domains of interest. Covariates that were significant at the 5% level were considered to influence the outcome.

Response criteria

Pain response was assessed by the best treatment response defined according to the European Society for Blood and Marrow Transplantation criteria and International Myeloma Working Group Uniform Response Criteria [10-12]. The relevant levels of response are defined as follows:

Very good partial response (VGPR)

This requires two consecutive assessments made at any time before the institution of any new therapy:

- Serum and urine M-component detectable by immunofixation but not on electrophoresis
- Objective response (OR)
- ≥90% reduction in serum M-component plus urine M-component <100 mg/24 h

Progressive disease (PD)

Increase of ≥25% from the lowest response value in any one or more of the following:

- Serum M-component (the absolute increase must be ≥0.5 g/dL)
- Urine M-component (the absolute increase must be ≥200 mg/24 h)
- Only in patients without measurable serum and urine M-protein levels: the difference between involved and uninvolved free light chain levels (the absolute increase must be >10 mg/dL)
- Bone marrow plasma cell percentage (the absolute percentage must be ≥10%)
- Definite development of new bone lesions or soft tissue plasmacytomas or definite increase in the size of existing bone lesions or soft tissue plasmacytomas

- Development of hypercalcemia (corrected serum calcium >11.5 mg/dL or

2.65 mmol/L) that can be attributed solely to the plasma cell proliferative disorder

Results

Table 4

MMRM estimates of overall treatment effect on BPI-SF domains

BPI-SF domain	Treatment	Estimate of effect Mean (SEM)	Estimated difference Mean (95% CI)	*p* value[a]
Pain Severity	ELd	0.717 (0.4076)	0.045 (−0.236, 0.325)	0.7548
	Ld	0.673 (0.3988)		
Pain Interference	ELd	1.121 (0.4746)	0.123 (−0.200, 0.447)	0.4543
	Ld	0.998 (0.4638)		
Worst Pain	ELd	0.862 (0.5220)	0.071 (−0.283, 0.424)	0.6945
	Ld	0.791 (0.5090)		

[a]Difference between treatment groups in overall change from baseline. *BPI-SF* Brief Pain Inventory–Short Form, *ELd* elotuzumab, lenalidomide, and dexamethasone, *Ld* lenalidomide and dexamethasone, *MMRM* mixed-model repeated-measures, *SEM* standard error of the mean

Table 5

Pain response analyses for Worst Pain by clinical response and treatment while on treatment

Covariates used in the MMRM	Treatment	No sustained improvement, n (%)	Sustained improvement, n (%)	Difference, % (95% CI)
Treatment	ELd	237 (74.5)	81 (25.5)	1.77 (−4.97, 8.51)
	Ld	235 (76.3)	73 (23.7)	
OR	Without	144 (87.8)	20 (12.2)	−16.80 (−23.30, −10.30)
	With	328 (71.0)	134 (29.0)	
Without OR	ELd	62 (92.5)	5 (7.5)	−8.00 (−17.60, 1.56)
	Ld	82 (84.5)	15 (15.5)	
With OR	ELd	175 (69.7)	76 (30.3)	2.79 (−5.49, 11.07)
	Ld	153 (72.5)	58 (27.5)	

Difference in sustained improvement is calculated as the proportion of patients with sustained improvement in category 1 (p1) minus the proportion in category 2 with sustained improvement (p2); 95% CI is calculated as 1.96 × SEM, where SEM (p1 − p2) = sqrt[SEM (p1) 2 + SEM (p2) 2] and SEM (p) = sqrt[p (1 − p)/n]. Percentages are row percentages and therefore use the row total as the denominator. *ELd* elotuzumab, lenalidomide, and dexamethasone, *Ld* lenalidomide and dexamethasone, *MMRM* mixed-model repeated-measures, *OR* objective response, *SEM* standard error of the mean

Figures

Fig. 1 CONSORT patient disposition flow diagram for ELOQUENT-2. Patients in the elotuzumab group received elotuzumab plus lenalidomide and dexamethasone. Patients in the control group received lenalidomide plus dexamethasone. [a]Patients with disease progression as primary reason for discontinuing treatment. From New England Journal of Medicine, Lonial S, Dimopoulos M, Palumbo A, White D, Grosicki S, Spicka I, Walter-Croneck A, Moreau P, Mateos MV, Magen H, Belch A, Reece D, Beksac M, Spencer A, Oakervee H, Orlowski RZ, Taniwaki M, Röllig C, Einsele H, Wu KL, Singhal A, San-Miguel J, Matsumoto M, Katz J, Bleickardt E, Poulart V, Anderson KC, Richardson P; ELOQUENT-2 Investigators. Elotuzumab Therapy for Relapsed or Refractory Multiple Myeloma. 373 (7):621-631. Copyright © (2015) Massachusetts Medical Society. Reprinted with permission. Available at: http://www.nejm.org/doi/full/10.1056/NEJMoa1505654

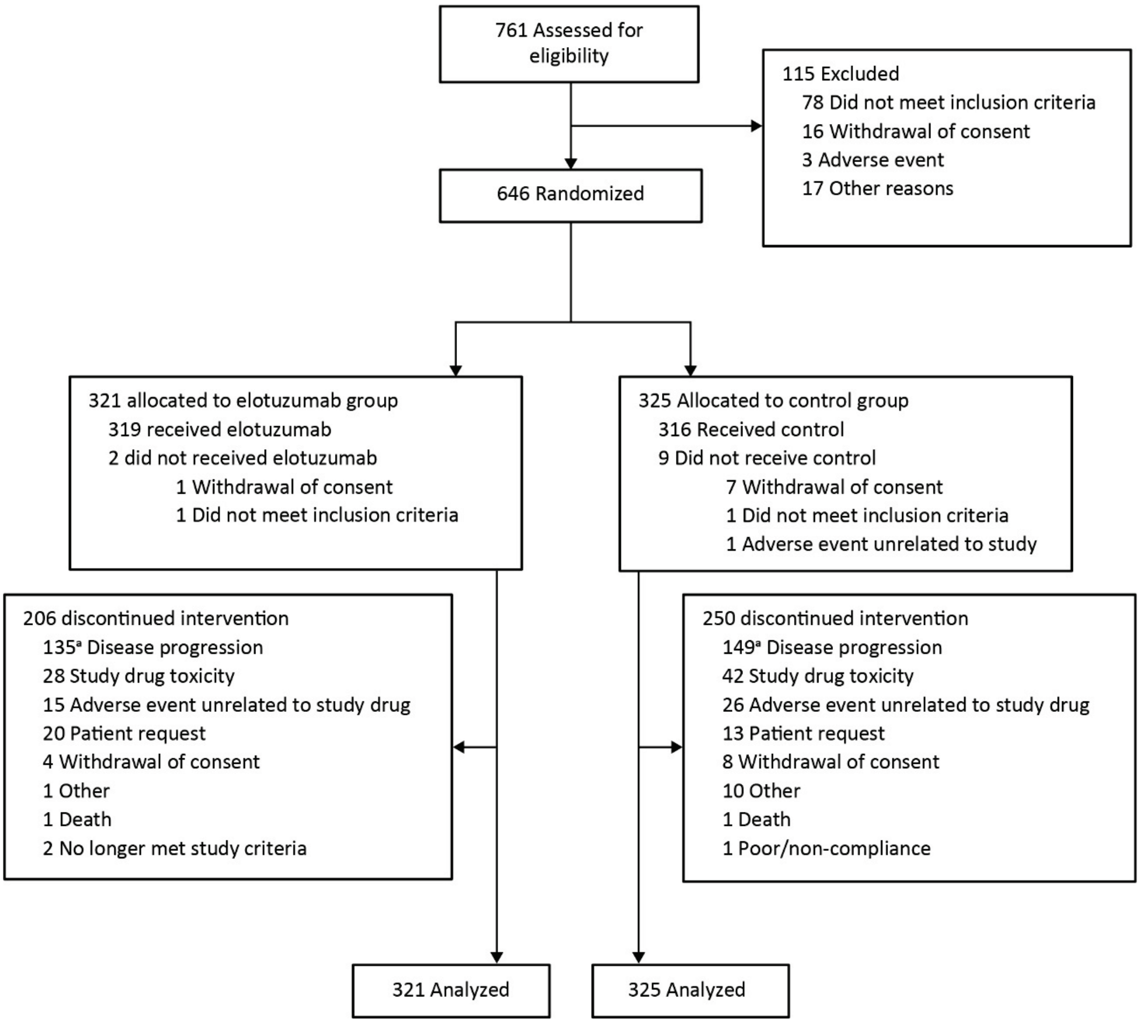

Fig. 2 Patient-reported pain during treatment: mean absolute values by treatment of **a** Pain Interference, and **b** Worst Pain. The dashed line indicates <30 patients per treatment group. *ELd* elotuzumab, lenalidomide, and dexamethasone, *EOS* end of study visit, *Ld* lenalidomide and dexamethasone

a

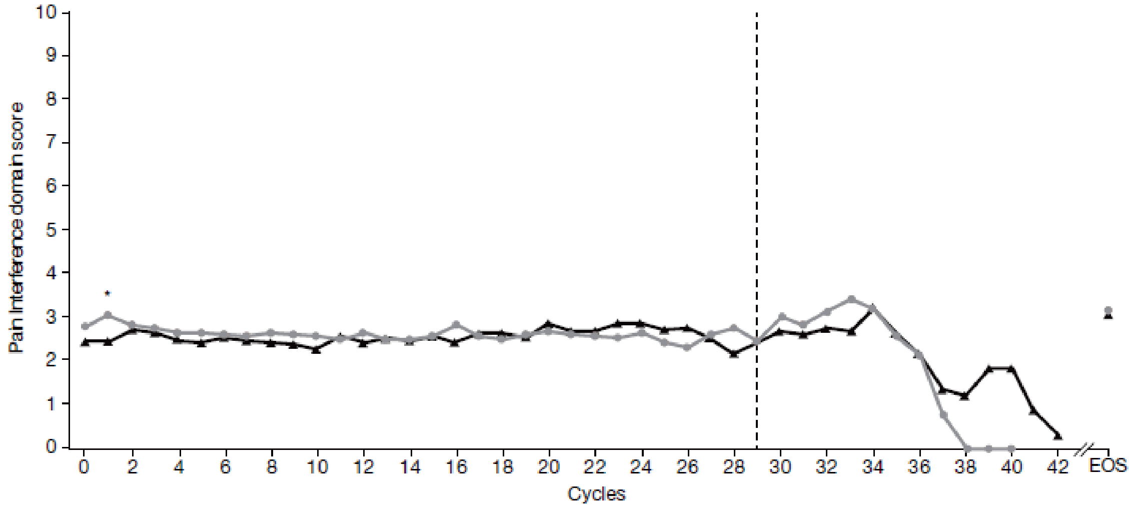

b

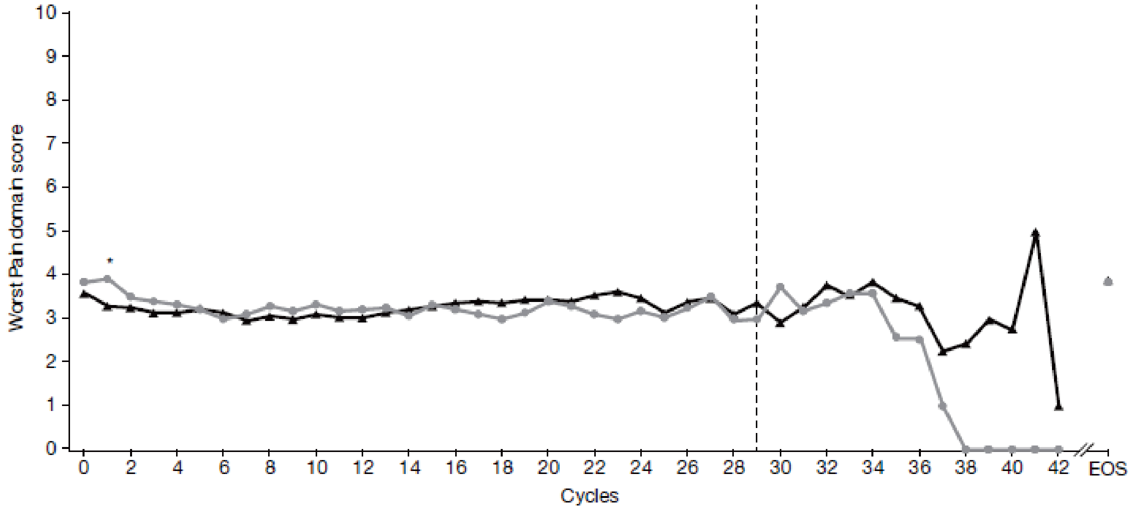

Fig. 3 Pain response during treatment, by clinical response: cumulative percentage of patients with a sustained improvement in Worst Pain among those who achieved, or did not achieve, an objective response to treatment by treatment. *ELd* elotuzumab, lenalidomide, and dexamethasone, *Ld* lenalidomide and dexamethasone, *OR* objective response

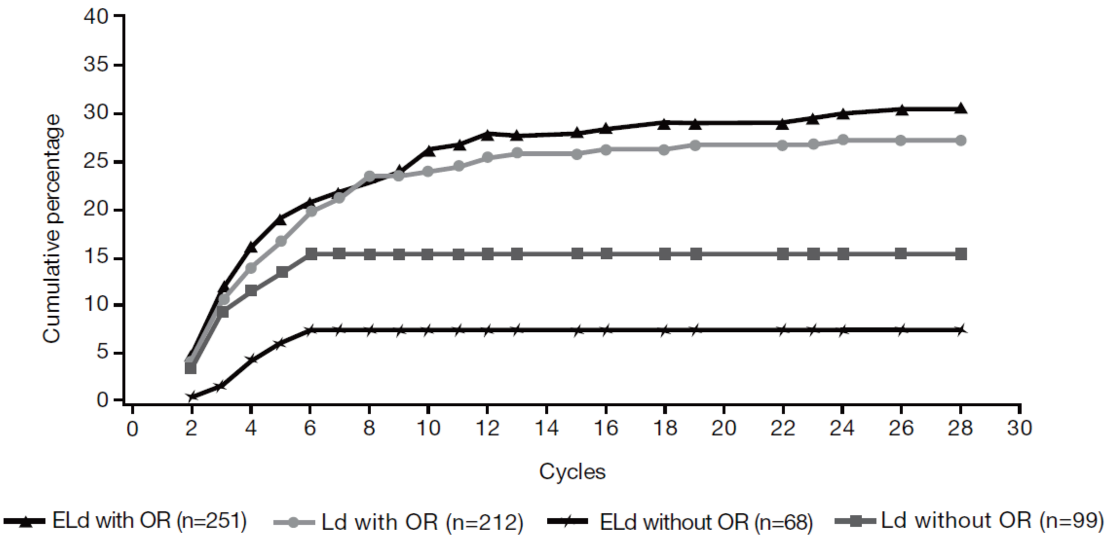

Fig. 4 Patient-reported pain during treatment, by best response to treatment: mean change from baseline in Pain Interference and Worst Pain scores in patients with a best response to treatment of at least a VGPR, less than a VGPR, or progressive disease. ELd elotuzumab, lenalidomide, and dexamethasone, Ld lenalidomide and dexamethasone, PD progressive disease, VGPR very good partial response

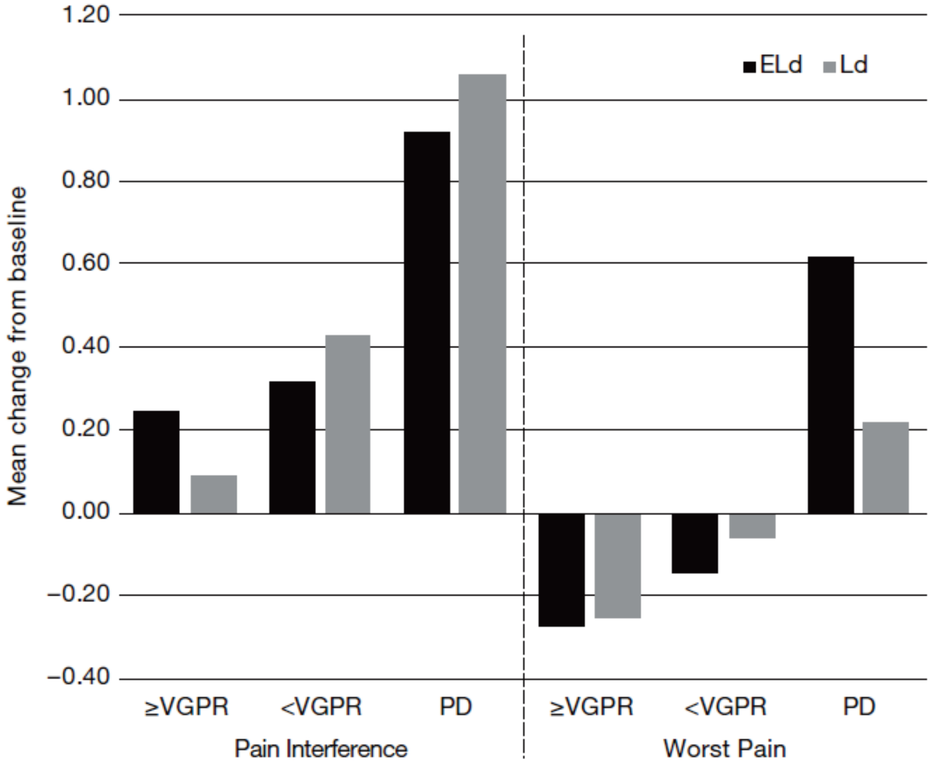

Fig. 5 Health-related quality of life during treatment, assessed by key domains of the EORTC QLQ-C30: mean absolute values by treatment for **a** Fatigue, **b** Physical Functioning, **c** Global Health Status/QoL, and **d** Pain. The dashed line indicates <30 patients per treatment group. Asterisks (*) denote statistical significance ($p < 0.05$) for the difference between treatments. *ELd* elotuzumab, lenalidomide, and dexamethasone, *EORTC QLQ-C30* European Organisation for Research and Treatment of Cancer Quality of Life Questionnaire–Core 30 module, *EOS* end of study visit, *Ld* lenalidomide and dexamethasone, *QoL* quality of life

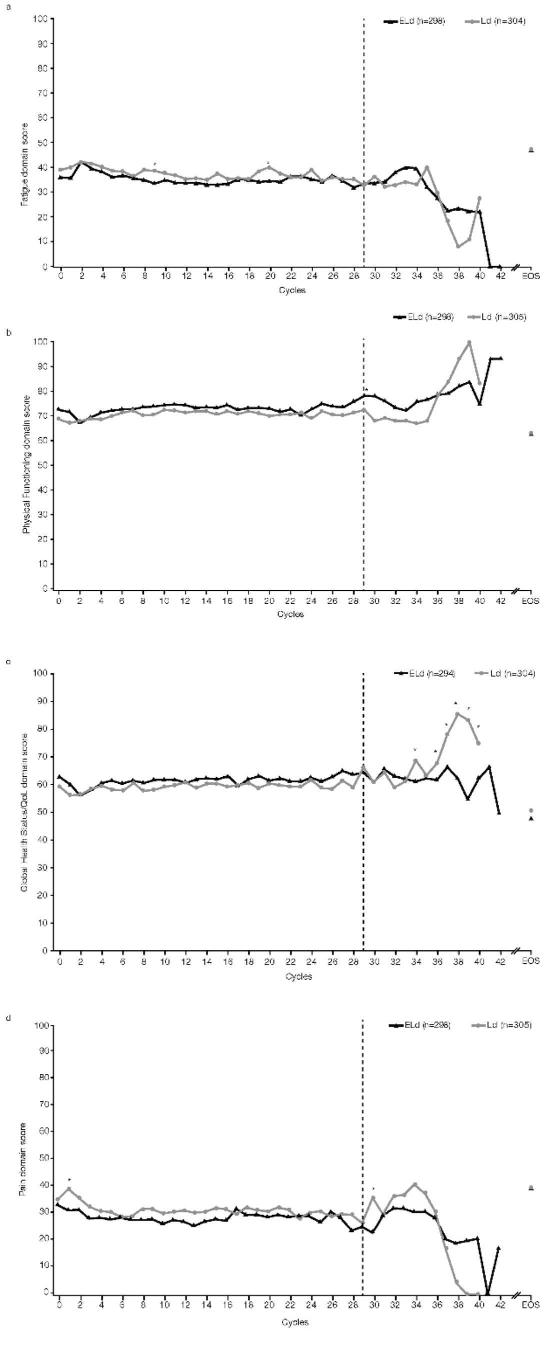

Fig. 6 Heath-related quality of life during treatment, assessed by key domains of the EORTC QLQ-MY20: absolute mean values by treatment for **a** Disease Symptoms and **b** Side Effects of Treatment. The dashed line indicates <30 patients per treatment group. *ELd* elotuzumab, lenalidomide, and dexamethasone, *EORTC QLQ-MY20* European Organisation for Research and Treatment of Cancer Quality of Life Questionnaire–myeloma-specific module, *EOS* end of study visit, *Ld* lenalidomide and dexamethasone

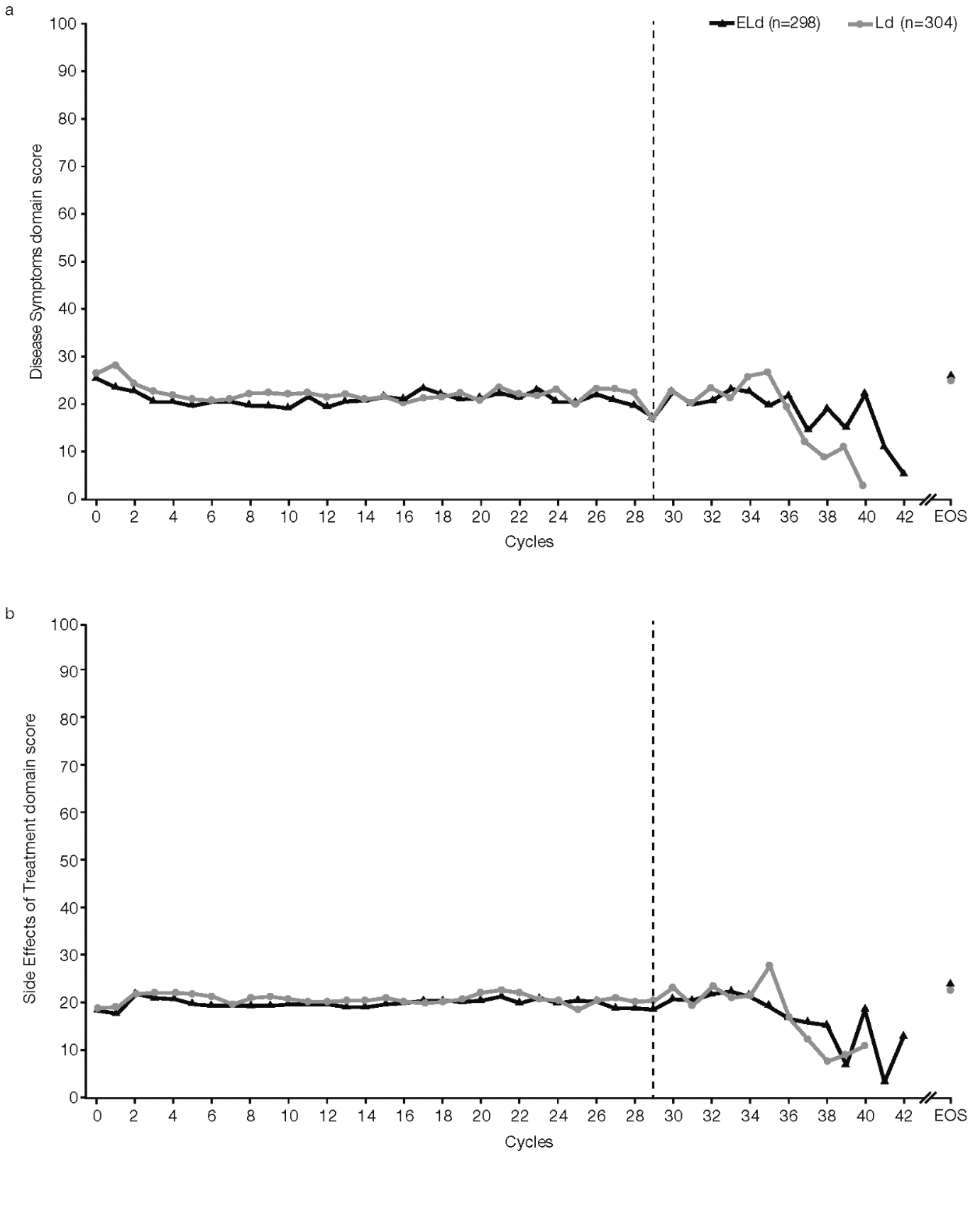

References

1. Cleeland CS, Ryan KM (1994) Pain assessment: global use of the Brief Pain Inventory. Ann Acad Med Singapore 23:129-138

2. Cleeland CS (2009 January 1) The Brief Pain Inventory User Guide. MD Anderson Cancer Center. https://www.mdanderson.org/documents/Departments-and-Divisions/Symptom-Research/BPI_UserGuide.pdf. Accessed 2016 Apr 11
3. Fayers P, Aaronson NK, Bjordal K, Curran D, Groenvold M, on behalf of the EORTC Quality of Life Study Group. EORTC QLQ-C30 Scoring Manual. Third edition. 2001. Brussels, EORTC Quality of Life Group.
4. Cocks K, Cohen D, Wisloff F, Sezer O, Lee S, Hippe E, Gimsing P, Turesson I, Hajek R, Smith A, Graham L, Phillips A, Stead M, Velikova G, Brown J (2007) An international field study of the reliability and validity of a disease-specific questionnaire module (the QLQ-MY20) in assessing the quality of life of patients with multiple myeloma. Eur J Cancer 43:1670-1678. doi: 10.1016/j.ecja.2007.04.022
5. Jaeschke R, Singer J, Guyatt GH (1989) Measurement of health status. Ascertaining the minimal clinically important difference. Control Clin Trials 10:407-415. doi: 10.1016/0197-2456(89)90005-6
6. Dimopoulos MA, Delforge M, Hajek R, Kropff M, Petrucci MT, Lewis P, Nixon A, Zhang J, Mei J, Palumbo A (2013) Lenalidomide, melphalan, and prednisone, followed by lenalidomide maintenance, improves health-related quality of life in newly diagnosed multiple myeloma patients aged 65 years or older: results of a randomized phase III trial. Haematologica 98:784-788. doi: 10.3324/haematol.2012.074534

7. Wyrwich KW, Norquist JM, Lenderking WR, Acaster S (2013) Methods for interpreting change over time in patient-reported outcome measures. Qual Life Res 22:475-483. doi: 10.1007/s11136-012-0175-x
8. Osoba D, Rodrigues G, Myles J, Zee B, Pater J (1998) Interpreting the significance of changes in health-related quality-of-life scores. J Clin Oncol 16:139-144. doi: 10.1200/JCO.1998.16.1.139
9. Cocks K, King MT, Velikova G, Martyn St-James M, Fayers PM, Brown JM (2011) Evidence-based guidelines for determination of sample size and interpretation of the European Organisation for the Research and Treatment of Cancer Quality of Life Questionnaire Core 30. J Clin Oncol 29:89-96. doi: 10.1200/JCO.2010.28.0107
10. Anderson KC, Kyle RA, Rajkumar SV, Stewart AK, Weber D, Richardson P (2008) Clinically relevant end points and new drug approvals for myeloma. Leukemia 22:231-239. doi: 10.1038/sj.leu.2405016
11. Blade J, Samson D, Reece D, Apperley J, Bjorkstrand B, Gahrton G, Gertz M, Giralt S, Jagannath S, Vesole D (1998) Criteria for evaluating disease response and progression in patients with multiple myeloma treated by high-dose therapy and haemopoietic stem cell transplantation. Myeloma Subcommittee of the EBMT. European Group for Blood and Marrow Transplant. Br J Haematol 102:1115-1123. doi: 10.1046/j.1365-2141.1998.00930.x
12. Durie BG, Harousseau JL, Miguel JS, Blade J, Barlogie B, Anderson K, Gertz M, Dimopoulos M, Westin J, Sonneveld P, Ludwig H, Gahrton G, Beksac M, Crowley J, Belch A, Boccadaro M, Cavo M, Turesson I, Joshua D, Vesole D, Kyle R, Alexanian R, Tricot G, Attal M, Merlini G, Powles R, Richardson P, Shimizu K, Tosi P, Morgan G, Rajkumar SV (2006) International uniform

response criteria for multiple myeloma. Leukemia 20:1467-1473. doi: 10.1038/sj.leu.2404284
